# Multitasking SecB chaperones in bacteria

**DOI:** 10.3389/fmicb.2014.00666

**Published:** 2014-12-05

**Authors:** Ambre Sala, Patricia Bordes, Pierre Genevaux

**Affiliations:** Laboratoire de Microbiologie et Génétique Moléculaire, Centre National de la Recherche Scientifique, UniversitéPaul Sabatier, Toulouse, France

**Keywords:** protein folding and targeting, SecA, DnaK, trigger factor, proteases, toxin–antitoxins

## Abstract

Protein export in bacteria is facilitated by the canonical SecB chaperone, which binds to unfolded precursor proteins, maintains them in a translocation competent state and specifically cooperates with the translocase motor SecA to ensure their proper targeting to the Sec translocon at the cytoplasmic membrane. Besides its key contribution to the Sec pathway, SecB chaperone tasking is critical for the secretion of the Sec-independent heme-binding protein HasA and actively contributes to the cellular network of chaperones that control general proteostasis in *Escherichia coli*, as judged by the significant interplay found between SecB and the trigger factor, DnaK and GroEL chaperones. Although SecB is mainly a proteobacterial chaperone associated with the presence of an outer membrane and outer membrane proteins, *secB*-like genes are also found in Gram-positive bacteria as well as in certain phages and plasmids, thus suggesting alternative functions. In addition, a SecB-like protein is also present in the major human pathogen *Mycobacterium tuberculosis* where it specifically controls a stress-responsive toxin–antitoxin system. This review focuses on such very diverse chaperone functions of SecB, both in *E. coli* and in other unrelated bacteria.

## PROTEIN FOLDING AND TARGETING IN BACTERIA

A major challenge for the cells is to ensure the proper folding and targeting of newly synthesized proteins to the different cellular compartments. Indeed, ongoing protein synthesis in the crowded cellular environment offers a window of opportunities for non-native interactions, which may eventually lead to proteostasis breakdown (Kramer et al., 2009). Therefore, to cope with noxious off pathways in protein biogenesis, cells have evolved universally conserved molecular chaperones and targeting factors, which act co- and/or post-translationally to guide the precise partitioning, localization and folding of newly synthesized proteins (Kramer et al., 2009; Kim et al., 2013).

In bacteria, the folding of newly synthesized proteins is mainly assisted by three highly conserved cytosolic chaperones, namely trigger factor (TF), DnaK/DnaJ/GrpE (DnaKJE), and GroEL/GroES (GroESL; Deuerling et al., 1999; Agashe et al., 2004; Kerner et al., 2005). The ribosome-bound TF is the first chaperone to interact co-translationally with most newly synthesized proteins (Valent et al., 1995). Although the majority of the cytosolic proteins can reach their native state following interaction with TF, a substantial amount of proteins (about 30%) need further co- and/or post-translational assistance by the downstream DnaKJE and GroESL chaperones (Bukau et al., 2000). Forceful genetic and biochemical analyzes have demonstrated significant overlap and cooperation between these three major chaperones, revealing a dynamic network of chaperones to control intracellular proteostasis (Teter et al., 1999; Genevaux et al., 2004; Calloni et al., 2012).

Targeting of newly synthesized proteins to the bacterial cytoplasmic membrane can occur either co- or post-translationally. While certain small membrane proteins are targeted post-translationally to the YidC insertase at the inner membrane (Dalbey et al., 2011), most integral membrane proteins as well as some presecretory proteins are targeted co-translationally by the ribosome-associated RNA-protein complex SRP (Saraogi and Shan, 2014). SRP binds to hydrophobic signal-anchor or signal sequence in nascent chains and targets them to the Sec translocon via interaction with its membrane receptor FtsY (Luirink and Sinning, 2004). The majority of presecretory proteins are translocated post-translationally either folded via the twin-arginine translocation (Tat) pathway or in a non-native state via the Sec pathway. The Tat system is known to translocate folded proteins or assembled protein complexes (up to 70 Å in diameter) through the cytoplasmic membrane. Tat substrate proteins possess an amino-terminal signal sequence with a conserved twin-arginine motif, which mediates post-translational targeting to the Tat translocon (Palmer and Berks, 2012; Patel et al., 2014). They are often assisted by specific cytosolic chaperones called redox enzyme maturation proteins (REMPs) and by the generic chaperones DnaK and GroEL, which likely prevent their degradation and premature export, and facilitate their assembly and functional interaction with the translocon (Castanie-Cornet et al., 2014).

The Sec translocon is conserved in all three domains of life. Its core is composed of a heterotrimeric membrane complex SecYEG in bacteria and Sec61αβγ in eukaryotes (du Plessis et al., 2011). While translocation in the endoplasmic reticulum via the Sec translocon is mainly mediated co-translationally and thus energized by polypeptide chain elongation, Sec translocation across the bacterial plasma membrane preferentially occurs post-translationally and energy is provided by the SecA ATPase motor component (Chatzi et al., 2013). In this case, SecA binds to presecretory proteins with mildly hydrophobic signal sequences, targets them to the Sec translocon at the inner membrane via a direct interaction with SecY, and subsequently drives the translocation process by successive cycles of ATP binding and hydrolysis.

Productive interaction with Sec is influenced by the folding rate of the substrate and facilitated by cytosolic chaperones capable of preventing premature folding, aggregation or degradation of precursor proteins (Randall and Hardy, 2002). Accordingly, all three main generic chaperone machines involved in *de novo* protein folding, namely TF, DnaKJE and GroEL, have been shown to participate at different levels in this process (Castanie-Cornet et al., 2014). Remarkably, most proteobacteria also possess the chaperone SecB, which in addition to its generic chaperone function has the ability to specifically interact with SecA to facilitate post-translational delivery of presecretory proteins to the Sec translocon (Bechtluft et al., 2010). This review describes the Sec-dependent and Sec-independent cellular functions of SecB, its interplay with other molecular chaperones as well as the distribution of SecB homologs in very diverse bacteria. The role of the recently identified SecB-like proteins in the control of intracellular stress-responsive toxin–antitoxin (TA) systems is also discussed.

## SecB AND THE Sec PATHWAY

SecB is a homotetrameric chaperone of 69 kDa with a cellular concentration estimated to be between 4 and 20 μM in *Escherichia coli*. SecB binds co- and/or post-translationally to non-native precursor proteins, maintaining them in competent state for delivery to the Sec translocon via a well-described interaction with its SecA partner (Randall and Hardy, 1995, 2002; Chatzi et al., 2013).

The *E. coli secB* gene was initially identified genetically by selecting for mutants that were defective in the export of a fusion protein composed of the N-terminal part of maltose-binding protein (MBP) preMBP (containing the signal sequence) and β-galactosidase (Kumamoto and Beckwith, 1983). Further experiments showed that *secB* mutations delayed or blocked the processing of a subset of preproteins and exhibited a synergistic effect with temperature-sensitive alleles of *secA*, thus revealing a role for SecB in export (Kumamoto and Beckwith, 1983, 1985). *E. coli secB* mutant strains were initially shown to be defective for growth on rich Luria broth media agar plates, but it later appeared that this phenotype was due to a polar effect on the downstream *gpsA* gene encoding a glycerol-3-phosphate dehydrogenase involved in phospholipid biosynthesis (Kumamoto and Beckwith, 1985; Shimizu et al., 1997). Deletion of *secB* without apparent polarity on *gpsA* results in a strong cold-sensitive phenotype below 23°C, a moderate temperature-sensitive phenotype at temperatures above 45°C and a hypersensitivity to several antibiotics (Ullers et al., 2007; **Table 1**). Most of the relevant phenotypes associated with *secB* mutations or SecB overexpression are shown in **Table 1**. Genetic interactions between *secB* and the various *p*rotein *l*ocalization *l*ocus (*prl)* mutations known to suppress the export defect of sequence signal deficient precursors are also presented (**Table 1**).

**Table 1:** Most relevant phenotypes associated with mutations or overexpression of the *E. coli* SecB chaperone.

SecB	Phenotypes^a^
*Single mutation*	
	Cs below 23°C and Ts at 46°C on LB agar plates^(1)^; sensitive to copper, ethanol, cholate, low pH, dibucaine, triclosan, verapamil, and to several antibiotics, including bacitracin, novobiocin, amoxicillin, carbenicillin, tetracycline, cefaclor, glufosfomycin, ceftazidime, tunicamycin^(2)^; partially resistant to phage U3^(3)^; produces slightly bigger cells^(4)^; and induces synthesis of heat-shock proteins^(4,5)^. Mutation in *secB* with polar effect on the downstream *gpsA* gene inhibits growth on LB agar plates^(6)^.
*Genetic interactions*	
	Mutation in *secB s*uppresses erythromycin and rifampin sensitivity of *lptE* mutants with increased outer-membrane permeability^(7)^; enhances growth and export defects of *secA51* mutation^(4)^; exacerbates Lon, DnaJ^(8)^ and TF^(1)^ toxicity. Ts, Cs and export defect of *secB* mutation are suppressed by *tig* mutation^(1)^; Cs is suppressed by *lon* mutation^(8)^ and by overexpression of σ^32(9)^, DnaK/DnaJ^(1,10)^, GroEL/GroES^(10,11)^, Rv1957^(12)^, SmegB^(13)^, and less efficiently by DnaJ259^(8)^ and SecA^(1)^. Export defect of *secB* mutations is partially suppressed by *secA853-128* mutation^(14)^. Synthetic lethal with *dnaKdnaJ* mutation^(1)^ and possibly with mutations in forty-one additional genes, including *groEL*, the *dsbC*, *lolA,* and *cpxP* genes encoding periplasmic stress proteins and/or chaperones, as well as *rplW* encoding for L23, the main chaperone docking site at the ribosomal peptide exit^(15)^. Likely presents positive or negative epistasis with eighty-nine additional mutations in cell envelope biogenesis genes^(15)^.
*Protein localization loci*	
	Mutation in *secB* blocks the phenotypic effects of the *prlC*8 (mutation in *opdA* encoding the cytoplasmic Oligopeptidase A) suppressor of *lamB* signal sequence mutation^(16)^; inhibits *prlA4* (*secY* [F286Y, I408N]) mediated suppression of maltose-binding protein (MBP) signal sequence mutations^(17,18)^ and *prlA4* and *prlZ1* mediated suppression of LamB signal sequence mutations^(19,20)^. The *prlA1001* (*secY* [I90N]) and *prlA1024* (*secY* [I408N]) mutations suppress export deficient maltose-binding protein in the absence of SecB^(17)^; the *prlF1* mutation in the antitoxin gene *sohA* of the SohA-YhaV toxin–antitoxin system suppresses SecB-dependent accumulation of LamB precursors^(21)^.
*Overexpression*	
	Partially suppresses the Ts of the double *dnaK tig* mutant^(22)^; affects expression of the cytoplasmic response regulator OmpR^(23)^; prevents activation of the mycobacterial HigBA1 toxin–antitoxin system expressed in *E. coli*^(12)^.

*^a^Phenotypes associated with mutation or overexpression of SecB, and genetic interactions between *secB* and other mutations. Cs and Ts stand for cold- and temperature-sensitive phenotype, respectively*.

*^1^Ullers et al. (2007); ^2^Nichols et al. (2011); ^3^Kumamoto and Beckwith (1985); ^4^Baars et al. (2006); ^5^Wild et al. (1993); ^6^Shimizu et al. (1997); ^7^Grabowicz et al. (2013); ^8^Sakr et al. (2010); ^9^Altman et al. (1991); ^10^Castanie-Cornet et al. (2014); ^11^Danese et al. (1995); ^12^Bordes et al. (2011); ^13^Sala et al. (2013b); ^14^Mcfarland et al. (1993); ^15^Babu et al. (2011); ^16^Trun and Silhavy (1989); ^17^Francetic et al. (1993); ^18^Derman et al. (1993); ^19^Wei and Stader (1994); ^20^Trun et al. (1988); ^21^Snyder and Silhavy (1992); ^22^Ullers et al. (2004); ^23^Jin and Inouye (1995)*.

**FIGURE 1:**
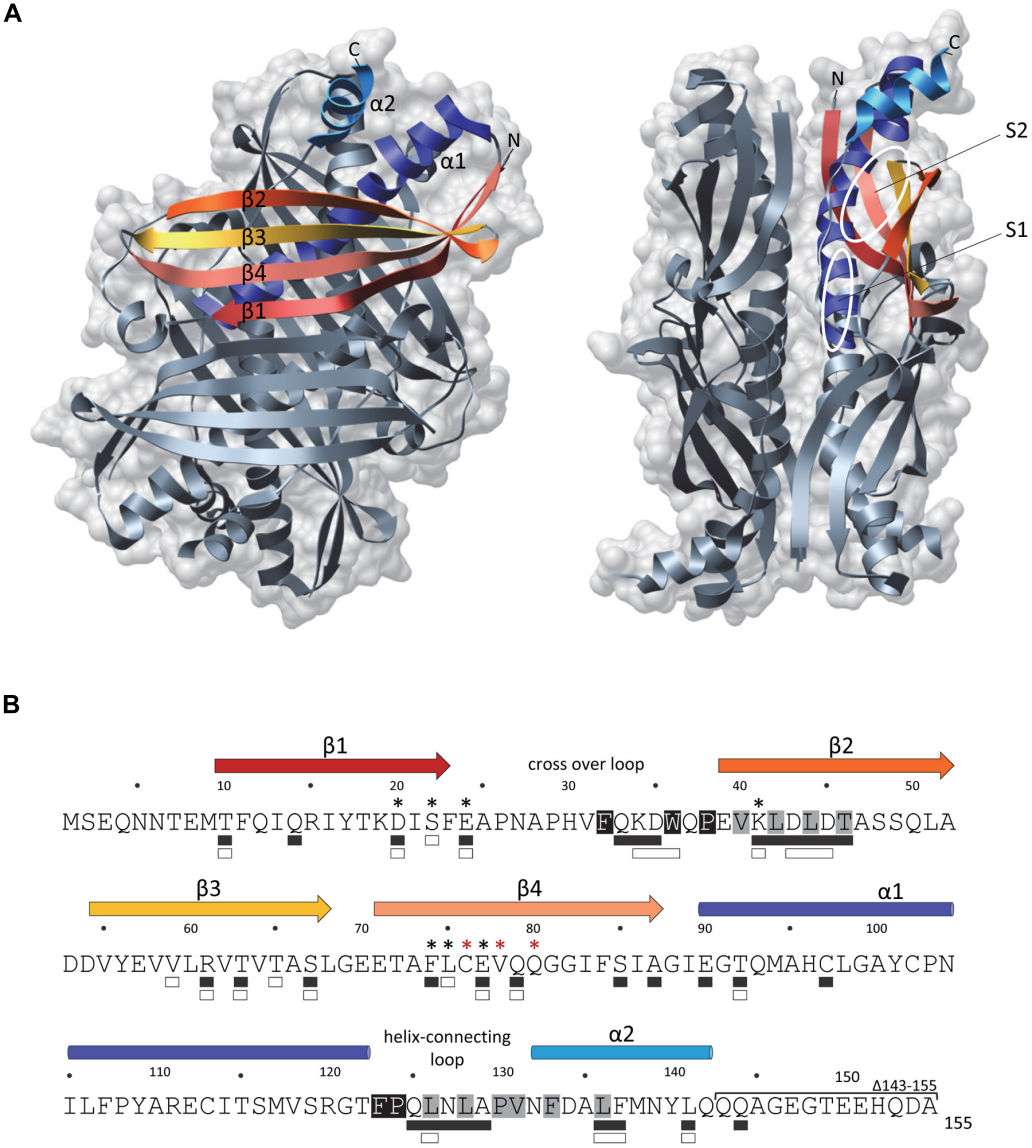
**The SecB chaperone. (A)** Three-dimensional structure of an *Escherichia coli* SecB tetramer (1QYN) in front (left) and side (right) views displayed in ribbon and molecular surface using Chimera. Secondary structural elements are colored in both representations as follows: β-sheet 1 in red, β-sheet 2 in orange, β-sheet 3 in yellow, β-sheet 4 in salmon, α-helix 1 in dark blue and α-helix 2 in light blue. On the side view, the proposed subsites 1 (S1) and 2 (S2) of interaction with the substrate are indicated. **(B)** The primary amino acid sequence of SecB is annotated with the secondary structural elements on the top with colors corresponding to **(A)**. Residues involved in the interaction with SecA or with the substrate are indicated below the sequence with white or black rectangles, respectively. Black asterisks indicate positions known to alter SecB function in export and red asterisks positions known to trigger aggregation of the protein. The C-terminal deletion 143–155 which alters the interaction with SecA is indicated. The residues predicted as being part of the subsites 1 and 2 of interaction with the substrate are highlighted in black and gray, respectively.

The crystal structures of SecB from *Haemophilus influenza* (Xu et al., 2000) and *E. coli* (Dekker et al., 2003) revealed that it forms a tetramer that assembles as a dimer of dimers (**Figure 1A**). SecB monomer is composed of four stranded antiparallel β-sheets (the first two strands being at opposite sides and connected by a cross over loop) and two α-helices separated by a helix connecting loop (**Figure 1A**). SecB dimer is formed via interactions between strands β1 and helices α1 of two monomers. The tetramer forms by packing the helices α1 of four monomers in between the eight stranded antiparallel β-sheets formed by each dimer, mainly via polar interactions. A peptide binding groove was suggested from these structures, lying between the end of the cross over loop and strand β2 on one side, and the helix connecting loop and the helix α2 on the other side. This proposed substrate binding region likely contains two subsites: the aromatic, deep subsite 1, and the shallower hydrophobic subsite 2, as presented in **Figure 1**. Two peptide binding grooves are present on each side of the SecB tetramer, each potentially allowing the binding of ∼20 amino acids-long extended segments. The fact that SecB can bind long fragments of approximately 150 residues in preprotein substrates (Khisty et al., 1995) suggests that these might wrap around the chaperone using several possible routes. Accordingly, electron paramagnetic resonance spectrometry analysis of spin labeled SecB variants in the presence of the physiologic SecB substrate galactose binding protein revealed that in addition to the proposed peptide binding groove, a much larger area of SecB appears to make contact with the substrate (Crane et al., 2006; **Figure 1B**).

SecB binds to non-native protein substrates with low specificity and high affinity (Kd in the nanomolar range), generally in a one to one ratio of tetrameric chaperone to substrate (Randall and Hardy, 1995). SecB binds to regions within the mature part of preprotein substrates and does not specifically recognize signal sequences (Gannon et al., 1989; Liu et al., 1989). Substrate selectivity by SecB is thought to occur via a kinetic partitioning between binding to the chaperone and folding, which is modulated by the affinity and the folding rate of the substrate protein (Hardy and Randall, 1991). Seminal work performed on the SecB substrate preMBP revealed the appearance of proteolysis resistant conformation of preMBP in the absence of SecB, thus suggesting that binding to SecB prevents precursor proteins from acquiring a stable tertiary structure incompatible with Sec-dependent translocation (Collier et al., 1988). A single molecule study recently confirmed that binding to SecB maintains preMBP in a molten globule-like state, preventing the formation of stable tertiary interactions (Bechtluft et al., 2007). SecB binding motif was identified by peptide scan of protein substrates as a nine amino acids-long segment enriched in aromatic and basic residues, with acidic residues strongly disfavored. Such motifs statistically occur every 20–30 amino acid residues in both exported and cytosolic proteins, thus suggesting low substrate specificity (Knoblauch et al., 1999).

Several SecB dependent presecretory substrates have been identified in *E. coli* by pulse chase experiments, sequence prediction or following analysis of protein aggregates that accumulate in the absence of the chaperone. This includes 25 presecretory proteins, namely CsgF, DegP, FhuA, FkpA, GBP, LamB, MBP, OmpA, OmpC, OmpF, OmpT, OmpX, OppA, PhoE, TolB, TolC, YagZ, YaiO, YbgF, YcgK, YfaZ, YgiW, YftM, YliI, and YncE (Hayashi and Wu, 1985; Kumamoto and Beckwith, 1985; Kusters et al., 1989; Laminet et al., 1991; Powers and Randall, 1995; Baars et al., 2006; Marani et al., 2006). Proteomic analyzes of protein aggregates that accumulate in a *secB* mutant also revealed the presence of a small number of aggregated cytosolic proteins (Baars et al., 2006; Sakr et al., 2010; see SecB Networking).

**FIGURE 2:**
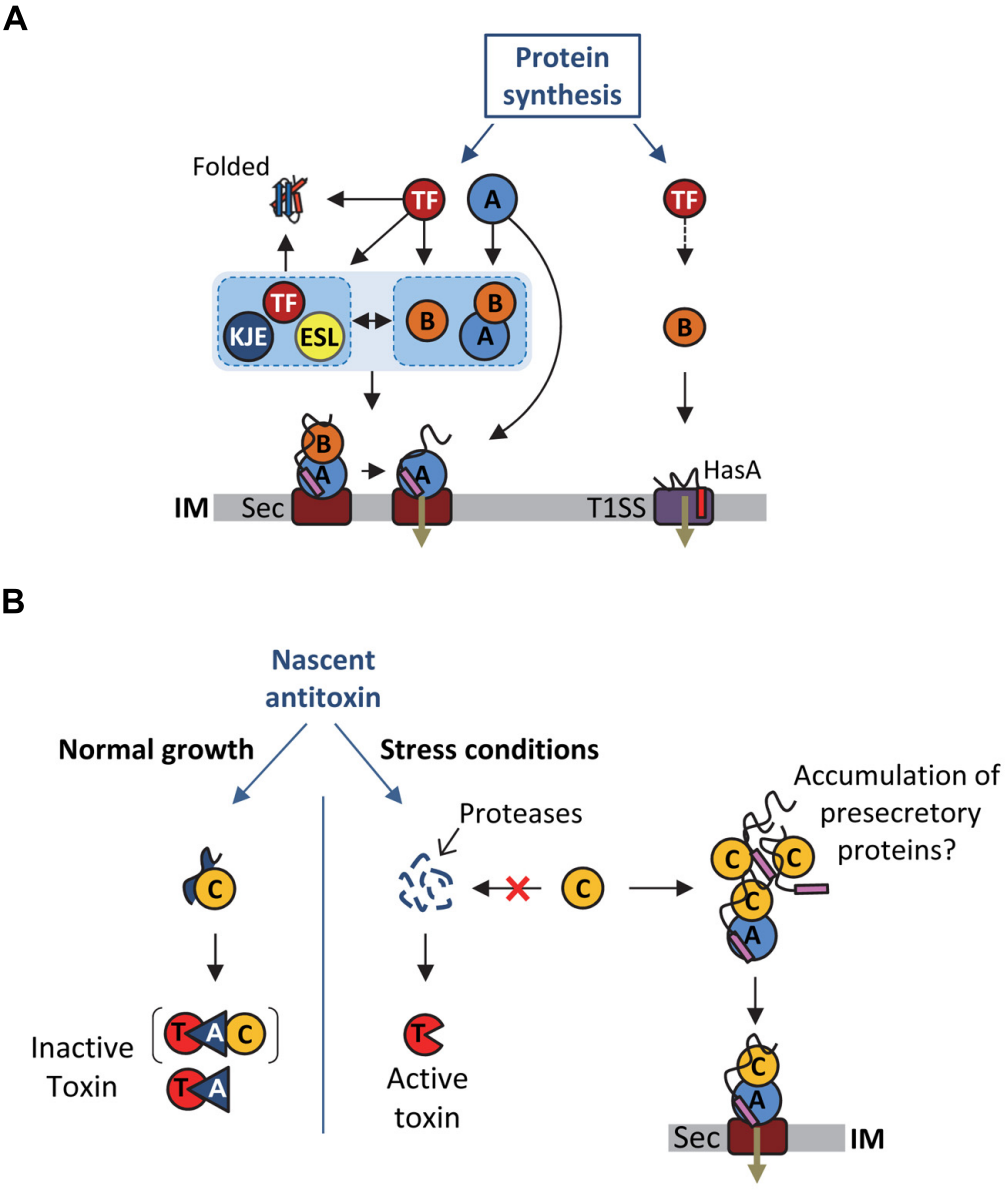
**Multiple functions of SecB chaperones. **(A)**** Proposed model for SecB-mediated protein targeting via the Sec pathway and the T1SS, and interplay between SecB and other generic chaperones. See text for details. Abbreviations for the chaperones and targeting factors presented are: trigger factor (TF), DnaK/DnaJ/GrpE (KJE), GroEL/GroES (ESL), SecB (B), SecA (A), type I secretion system (T1SS), SecYEG (Sec). IM stands for inner membrane. The T1SS and secretion signals are shown in red and purple, respectively. A black arrow indicates an interaction of the substrate with the chaperone or targeting factor and the black dashed arrow indicates a possible interaction of TF with T1SS substrates that was not yet investigated. **(B)** Proposed model for Rv1957 function in TA control. The different proteins are depicted as follows: toxin (T), antitoxin (A, blue triangle), Rv1957 chaperone (C), SecA1 (A, blue circle), SecYEG (Sec). IM stands for inner membrane. The signal sequence of presecretory proteins is showed in purple. The brackets indicate that it is not known yet whether the chaperone is part of the inactive complex. The red cross indicates that under certain stress conditions the chaperone could be recruited to rescue accumulating presecretory proteins. In this case, the chaperone would no longer be available to protect the antitoxin from degradation by proteases and to facilitate its interaction with the toxin, thus provoking toxin activation and bacterial growth inhibition until normal condition resume.

As stated above, SecB directly targets presecretory proteins to the Sec pathway via its specific interaction with the peripheral ATPase SecA: the motor component of the Sec translocon (Hartl et al., 1990; **Figure 2A**). SecA is an essential cytosolic protein of 102 kDa with an estimated cellular concentration of ∼7 μM in *E. coli* (Kusters et al., 2011; Chatzi et al., 2013). SecA forms a homodimer in solution, is found either soluble or membrane-bound, and can specifically interact with translating ribosomes mainly via its N-terminal helix (Singh et al., 2014). SecB can interact with both membrane-bound and soluble SecA, albeit with a significantly lower affinity for soluble SecA (1.5 μM versus 30 nM Kds, respectively; den Blaauwen et al., 1997). Interaction between SecB and membrane-bound SecA is further increased in the presence of precursor proteins (Kd of ∼10 nM), in order to facilitate the targeting of precursor proteins to the translocon (Fekkes et al., 1997).

Contact regions between SecB and SecA have been studied as well. SecB mutants with amino acid substitutions at positions D20, E24, L75, and E77 that were originally selected on the basis of their export defect (Gannon and Kumamoto, 1993; Kimsey et al., 1995), were later shown to be defective for binding to SecA (Woodbury et al., 2000). Accordingly, the crystal structures of SecB revealed that all these residues localize within the negatively charged surface formed by the β-sheets on both sides of the tetramer (**Figure 1**; Xu et al., 2000; Dekker et al., 2003). The main SecB binding site of SecA, which encompasses the last 22 C-terminal amino acid of SecA is highly enriched in basic residues and possesses a zinc binding site required for a functional interaction with SecB (Fekkes et al., 1998, 1999). The structure of *H. influenzae* SecB in complex with the last 24 amino acids of SecA further established such a specific binding occurring mainly through electrostatic interactions, with one SecA C-terminal peptide being bound to each β-sheet surface on both sides of a SecB tetramer (Zhou and Xu, 2003). This is consistent with a model in which one SecA dimer binds to one SecB tetramer (Randall et al., 2005). An additional contact site between the two proteins has been described, which consists of the C-terminal α-helices of SecB and the N-terminal part of SecA involved in dimerization and ribosome binding (Randall et al., 2004, 2005; Singh et al., 2014). Such interaction was proposed to trigger dissociation of the SecA dimer, thus allowing the opening of a peptide binding groove that would favor substrate transfer from SecB to SecA (Randall et al., 2005). These two surfaces of contact were confirmed by spin-labeling analyzes of SecB upon SecA binding (Crane et al., 2005). Interestingly, this work also showed that the surfaces of SecB that interact with the precursor and with SecA significantly overlap, thus likely facilitating substrate transfer for translocation (**Figure 1B**; Crane et al., 2005). Efficient transfer of the precursor protein from SecB to SecA requires both a correct interaction with SecB and the binding of the functional signal sequence to SecA, which has a strong affinity for signal sequences (Fekkes et al., 1998). To date, the precise mechanism of substrate transfer remains unknown. Once the ATP-dependent translocation process initiates, SecB is released from the Sec translocon and is now free to initiate a new cycle of binding to precursor proteins (Fekkes et al., 1997). The fact that SecB has the ability to stimulate SecA ATPase activity suggests that it could contribute to the translocation initiation process as well (Miller et al., 2002).

In addition to the post-translational targeting of the SecB-precursor complex to the SecYEG-bound SecA, recent studies suggest that SecB might be directly recruited to the preformed cytosolic SecA-precursor complex prior to SecA interaction with the protein conducting channel SecYEG (Chatzi et al., 2013; **Figure 2A**). This model is supported by the recently described interaction of SecA with the L23 ribosomal protein platform for ribosome interacting factors at the ribosome exit tunnel (Huber et al., 2011; Singh et al., 2014). Together these data further highlight the multifaceted interaction between SecA and SecB, and its key contribution to the selective post-translational targeting of precursor proteins in *E. coli*.

## SecB AND TYPE 1 SECRETION SYSTEMS

Besides its chaperone function during protein export via the Sec pathway SecB is a key player in the secretion of the small Sec-independent HasA hemoprotein (19.3 kDa), which is part of the heme acquisition system of *Serratia marcescens* (Letoffe et al., 1994). So far, HasA is the only known type 1 secretion system (T1SS) substrate that is strictly dependent on SecB. The T1SS, which is widespread among Gram-negative bacteria directs the one step translocation of polypeptides across both the inner and outer membranes, directly to the extracellular medium (Delepelaire, 2004). It allows secretion of proteins of diverse sizes (19–800 kDa) and functions (toxins, lipases, heme-binding, or S-layer proteins), which are presumably transported in an unfolded state via a C-terminal uncleaved secretion signal (Delepelaire, 2004; Holland et al., 2005). HasA of *Serratia marcescens* is secreted by an archetypal T1SS comprising an inner membrane ABC (ATP binding cassette) protein HasD, a periplasmic adaptor HasE, and an outer membrane channel-forming protein of the TolC family, named HasF (Letoffe et al., 1994).

SecB interacts with nascent HasA early during synthesis and holds it in an unfolded conformation competent for productive interaction with the ABC transporter HasD at the inner membrane (**Figure 2A**; Delepelaire and Wandersman, 1998; Debarbieux and Wandersman, 2001). In support of such antifolding activity of SecB, it has been shown that slow folding mutants of HasA are secreted independently of SecB (Wolff et al., 2003). Despite the fact that SecB allows a functional interaction between the N-terminal region of HasA and HasD, no direct interaction could be detected between SecB and the transporter (Sapriel et al., 2002, 2003; Wolff et al., 2003). Remarkably, point mutations in SecB that are known to affect its interaction with SecA (i.e., mutations D20A, E24A, L75R, and E77V; **Figure 1B**) exhibited very little or no effect on HasA secretion, thus indicating that SecB functions independently of SecA in this process. In contrast, SecB mutations affecting its oligomeric state and thus its chaperone function (mutations C76Y and Q80R; Kimsey et al., 1995; Muren et al., 1999) have a very strong effect on HasA secretion (Sapriel et al., 2003), suggesting that substrate binding by SecB is sufficient in this case. To date, the use of SecB generic chaperone function by SecA-independent secretion systems has only been shown for HasDEF and it remains to be determined whether other systems require similar assistance by SecB, and to what extent such chaperone redeployment could affect proper functioning of the SecA/SecB cascade *in vivo*.

## SecB NETWORKING

Significant interplay between SecB and other major cytosolic chaperones has been described (Castanie-Cornet et al., 2014; **Figure 2A**). The functional cooperation and/or overlap, as well as the strong genetic interactions observed between SecB, TF and DnaKJE suggest a key role for SecB as part of the chaperone network that orchestrates proper protein folding and targeting in *E. coli*. Albeit significantly less studied, a discrete link between SecB and the chaperonin GroEL has also been shown in some cases. In this part, we describe the intricate relationship between SecB and these main chaperones and discuss how SecB chaperone tasking contributes to such proteostasis network.

### SecB AND THE RIBOSOME-BOUND TRIGGER FACTOR CHAPERONE

The TF chaperone interacts with most newly synthesized polypeptides in *E. coli* (Valent et al., 1995). It is believed that about 70% of the *E. coli* cytosolic proteins interacting with TF reach their native state without further assistance (Deuerling et al., 1999; Teter et al., 1999). TF specifically binds to the ribosomal protein L23 in the vicinity of the polypeptide exit tunnel and cycles on and off the ribosome in an ATP-independent manner (Kramer et al., 2002; Ferbitz et al., 2004; Genevaux et al., 2004). Following release from the ribosome, TF can stay bound to elongating polypeptides and facilitate substrate transfer to downstream chaperones or possibly to the Sec translocon (Crooke et al., 1988b; Kaiser et al., 2006; Raine et al., 2006; Hoffmann et al., 2010; Saio et al., 2014). TF can delay the folding of large proteins and exhibits unfolding activity (Agashe et al., 2004; Hoffmann et al., 2012; O’Brien et al., 2012), which may facilitate targeting of presecretory proteins to the Sec translocon, as observed for SecB. TF interacts with outer membrane proteins (OMPs) and several OMPs and periplasmic proteins are significantly decreased in the absence of TF (Oh et al., 2011). Remarkably, a substantial number of these exported subtrates is shared between SecB and TF: this includes precursors of OmpA, OmpC, OmpF, LamB, PhoE, TolC, DegP, FkpA, OppA, Bla, and MBP (Castanie-Cornet et al., 2014). Yet, in contrast with SecB, a direct role for TF in stabilizing translocation competent precursors has only been shown for proOmpA and in this case, deletion of the *tig* gene encoding TF exhibited no significant defect on proOmpA processing (Crooke and Wickner, 1987; Crooke et al., 1988a,b). Instead, *tig* mutation was shown to accelerate translocation of several known SecB substrates, namely OmpA, OmpC, and OmpF (Lill et al., 1988; Guthrie and Wickner, 1990; Lee and Bernstein, 2002; Genevaux et al., 2004; Ullers et al., 2007) and to fully suppress both cold-sensitive and temperature-sensitive phenotypes of a *secB* null strain (**Table 1**; Guthrie and Wickner, 1990; Lee and Bernstein, 2002; Genevaux et al., 2004; Ullers et al., 2007). These data suggest that ribosome-bound TF could facilitate post-translational targeting of precursors by maintaining them competent either for binding to membrane-bound SecA (Gouridis et al., 2009) or for transfer to SecB, DnaKJE, or GroESL (**Figure 2A**; see subsections below). The fact that both TF and SecA bind to L23 at the ribosomal polypeptide exit suggests that TF could either cooperate with SecA or prevent unproductive SecA binding to precursors that first need to transit via SecB (**Figure 2**; Karamyshev and Johnson, 2005; Huber et al., 2011; Singh et al., 2014). Although more work is needed to shed light on such possible interplays between SecA, TF and SecB, it is important to note that both *secB* and *rplW* (the gene encoding L23) mutations likely synergize *in vivo*, further supporting an important role for SecB in this process (**Table 1**).

### SecB AND THE DnaKJE CHAPERONE MACHINE

The ATP-dependent chaperone DnaK of *E. coli* is a well-characterized member of the Hsp70 chaperone family. It is an abundant cytosolic chaperone expressed constitutively and induced in response to different stresses (Genevaux et al., 2007). The DnaK chaperone cycle is tightly regulated by essential co-chaperones: (i) the DnaJ (Hsp40) co-chaperone family members that stimulate DnaK’s weak ATP activity and facilitate substrate delivery to DnaK, and (ii) the nucleotide exchange factor GrpE, which mediates the dissociation of ADP and the subsequent binding of a new ATP that triggers substrate release from DnaK (Liberek et al., 1991; Harrison et al., 1997; Brehmer et al., 2001). DnaK preferentially interacts with short extended hydrophobic polypeptide sequences accessible during *de novo* protein folding, translocation through biological membranes, during stress or within native protein complexes (Rudiger et al., 1997). In agreement with such a variety of potential interactors, the recently described *in vivo* interactome of DnaK obtained in the presence of SecB revealed that DnaK interacts with more than six hundred *E. coli* proteins at 37°C, including cytosolic (∼80%), inner membrane (∼11%), outer membrane (∼3%) and periplasmic proteins (∼3%; Calloni et al., 2012).

Most of our current knowledge concerning DnaKJE’s contribution to the Sec pathway originates from studies concerning *secB* mutants and/or SecB substrates (Castanie-Cornet et al., 2014). Indeed, it has been shown that export of the SecB substrates OmpA, OmpC, and OmpF strongly relies on DnaK when protein translocation is compromised (Qi et al., 2002), and that overexpression of DnaKJ suppresses both the cold-sensitive phenotype of a *secB* null strain and the export defect of the SecB-dependent substrates LamB and MBP (Wild et al., 1992; Ullers et al., 2007; Castanie-Cornet et al., 2014). Although, export of both LamB and MBP is not affected by a *dnaK* mutation (Wild et al., 1992), depletion of DnaKJ in the absence of SecB showed a further decrease in the processing of these proteins and a robust accumulation of protein aggregates in the *E. coli* cytoplasm (Wild et al., 1992; Ullers et al., 2007). These aggregated proteins include known DnaK substrates and several OMPs (i.e., OmpA, OmpC, OmpX, and PhoE) previously known as SecB substrates (Ullers et al., 2007). Such a major overlap between these two chaperones is further supported by the fact that SecB substrates were recently identified as *bona fide* DnaK interactors *in vivo* (Calloni et al., 2012). This includes the OMPs OmpA, OmpC, OmpF, OmpT and OmpX, and the periplasmic proteins OppA and DegP. Accordingly, peptide binding scans revealed that SecB and DnaK share many potential binding sites in polypeptide substrates and could interact with similar regions within protein (Knoblauch et al., 1999). These data are in complete agreement with the fact that mutations in *secB* and *dnaK* (or *dnaJ*) exhibit synthetic lethality (**Table 1**), and that expression of DnaK is upregulated in the absence of SecB, and reciprocally (Muller, 1996; Ullers et al., 2007). These data also suggest that both chaperones could work in concert to assist the post-translational translocation of certain Sec substrates (Sakr et al., 2010). The physical interaction recently found between SecB and DnaK *in vivo* is in agreement with such hypothesis (Calloni et al., 2012).

### SecB AND THE TF/DnaK PATHWAY FOR CYTOSOLIC PROTEIN FOLDING

In addition to protein export, a role for SecB in rescuing cytosolic protein folding has been proposed. Such a SecB function has emerged from studies generally focusing on both TF and DnaK chaperones. Indeed, it has been shown that SecB overexpression efficiently rescues the severe growth defect of a chaperone-deficient strain carrying both *dnaK* and *tig* mutations, and suppresses the DnaK/TF-dependent accumulation of aggregated cytosolic proteins (Ullers et al., 2004). *In vitro* cross-linking experiments further showed that SecB is indeed capable of interacting co- and/or post-translationally with nascent RpoB in the absence of both chaperones. Such a possible SecB function is further supported by the fact that (i) SecB has preference for unstructured stretches of polypeptides that are not specifically found in exported proteins (Knoblauch et al., 1999), (ii) SecB prevents luciferase aggregation and cooperates with DnaKJE in the refolding of luciferase *in vitro* (Knoblauch et al., 1999), and (iii) cytosolic proteins can be isolated from aggregated protein fractions in both *secB* and *secB lon* mutant strains (Baars et al., 2006; Sakr et al., 2010). More work is warranted to elucidate whether SecB indeed has cytosolic protein substrates *in vivo*. Of note, the SecB-like chaperone Rv1957 in *Mycobacterium tuberculosis* was shown to directly assist the folding of a cytosolic antitoxin, arguing for such possible SecB function in other bacteria (see part below).

### SecB AND THE CHAPERONIN GroESL

The third main molecular chaperone potentially linked to SecB in *E. coli* is the chaperonin GroESL. The ATP-dependent chaperonin GroEL is a well-characterized member of the Hsp60 chaperone family. Together with its co-chaperone GroES (Hsp10), it provides both a protected environment and a functional assistance to polypeptides generally up to 60 kDa. GroEL forms a barrel-shaped complex composed of two heptameric rings assembled back-to-back (Saibil et al., 2013). The GroEL folding cavity can be closed by a seven GroES co-chaperone lid, which allows confinement of the polypeptide. It is believed that GroESL interacts with more than 10% of the *E. coli* cytosolic proteins, including aggregation-prone proteins that are strictly chaperonin-dependent for their folding *in vivo* (Kerner et al., 2005).

Although poorly investigated, a direct involvement of GroESL in the Sec pathway has been observed and several SecB substrates have been shown to interact with or to be processed by GroEL (Kusukawa et al., 1989; Lecker et al., 1989; Phillips and Silhavy, 1990). Remarkably, five known SecB substrates were recently identified as GroEL interactors *in vivo*. These include three OMPs, namely OmpA, OmpC and OmpF, and two periplasmic proteins OppA and YncE (Watanabe et al., 1988; Kerner et al., 2005; Baars et al., 2006). In addition, GroEL was previously shown to interact with prePhoE and proOmpA *in vitro*, and to stabilize proOmpA for translocation (Kusukawa et al., 1989; Lecker et al., 1989). Although *groESL* mutations exhibit no apparent effect on proOmpA and proOmpF processing, overexpression of GroESL efficiently rescues the cold-sensitive phenotype of a *secB* null strain (unpublished data). The fact that endogenous SecB level also increases in strains with impaired GroESL is in agreement with such findings (Muller, 1996). Together these data suggest that GroESL may actively contribute to the Sec-dependent export process, perhaps rescuing SecB substrates under certain stresses or even cooperate with SecB to facilitate their transfer to SecA, as proposed for TF and DnaK.

## SecB-LIKE CHAPERONES AND TOXIN–ANTITOXIN SYSTEMS

As stated above, SecB is usually found in proteobacteria. Yet, some SecB-like sequences are also found in other taxonomic groups, including Gram-positive bacteria (Sala et al., 2013b). The major human pathogen *M. tuberculosis* also encodes a SecB-like protein, namely Rv1957, which shares 19% amino acid sequence identity with the *E. coli* SecB. The fact that mycobacteria have a well-defined and characteristic outer membrane, named the mycomembrane, with a significant number of predicted OMPs suggests that these bacteria could make use of such SecB chaperone function for the targeting of their OMPs to the Sec translocon (Zuber et al., 2008; Mah et al., 2010). Previous work showed that Rv1957 can replace SecB export function in *E. coli*, partially restoring the processing of both proOmpA and preMBP, and complement the cold-sensitive phenotype of a *secB* mutant strain (**Table 1**). *In vitro*, Rv1957 also forms a tetramer in solution and efficiently prevents aggregation of the known *E. coli* SecB substrate proOmpC at a level comparable to that of SecB (Bordes et al., 2011). These results strongly suggest that Rv1957 could act as a *bona fide* SecB chaperone to assist protein export in *M. tuberculosis*.

In contrast with *E. coli* SecB, the Rv1957 encoding gene is clustered together with genes that are part of a stress-responsive type II TA system related to the HigBA family (Host Inhibition of Growth; Gupta, 2009; Ramage et al., 2009; Bordes et al., 2011; Sala et al., 2013a,b; Schuessler et al., 2013). Type II TA systems are genetic modules composed of a stable toxin and a less stable antitoxin, which interact together to form a complex in which the toxin is inactive (Gerdes et al., 2005; Goeders and Van Melderen, 2014). Under specific stress condition, the antitoxin is degraded by activated proteases, provoking the release of the active toxin, which will then act on its intracellular targets. Toxins from type II TA generally target essential cellular functions, such as translation or replication, resulting in growth inhibition. Modulation of bacterial growth by TA systems in response to environmental insults likely favors adaptation to stress (Lewis, 2010; Yamaguchi and Inouye, 2011). Remarkably, the SecB-like chaperone Rv1957 from *M. tuberculosis* specifically controls the inhibition of the HigBA TA system (**Figure 2B**). Indeed, Rv1957 interacts directly with the HigA antitoxin and protects it from both aggregation and degradation by proteases, thus facilitating its folding and subsequent interaction with the toxin. This chaperone function is necessary for the efficient inhibition of the toxin by the antagonistic antitoxin (Bordes et al., 2011). Such a tripartite system, named TAC for toxin-antitoxin-chaperone, is the first example of a TA system controlled by a molecular chaperone.

The hypothetic dual role of Rv1957 both as a generic chaperone potentially assisting protein export and as a specialized chaperone controlling a bacterial growth tuning system raises the question of a possible link between these two functions under certain conditions (**Figure 2B**). An attractive hypothesis is that in case of a compromised translocon accumulation of preproteins could compete with the antitoxin for Rv1957 binding, resulting in antitoxin degradation and subsequent toxin activation. In this model, the SecB-like chaperone would thus function as a molecular sentinel to watch over protein export. The fact that the presence of a *secB* open reading frame associated with TA modules is not unique to *M. tuberculosis* or to mycobacteria (Sala et al., 2013b) indicates that such a mechanism might be conserved (see part below).

## TAXONOMIC DISTRIBUTION OF SOLITARY AND TA-ASSOCIATED SecB

**FIGURE 3:**
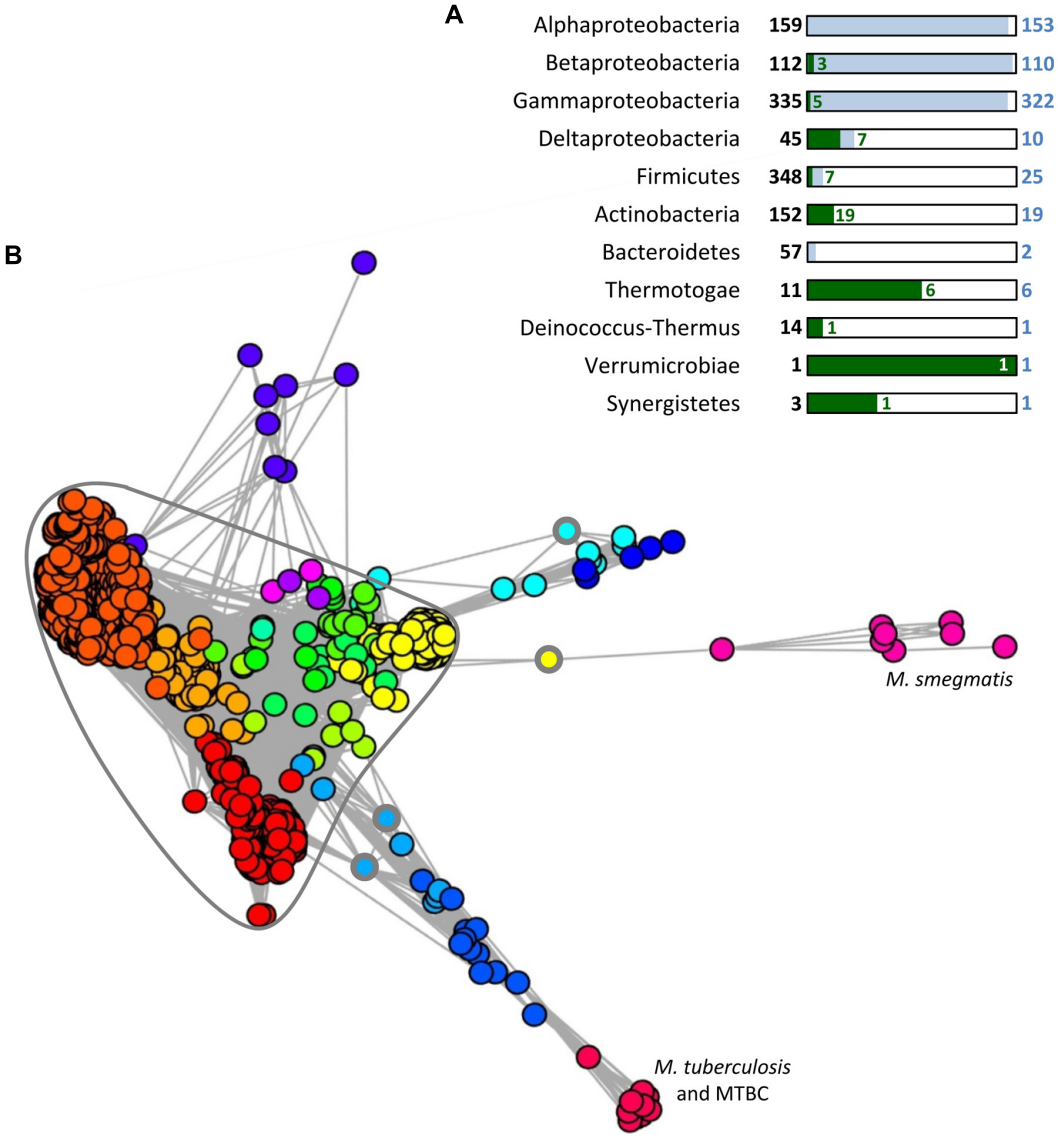
**Distribution and conservation of SecB sequences in bacteria. (A)** Taxonomic distribution of SecB sequences. A number of 1631 complete and cured bacterial genomes (local database) were analyzed for the presence of SecB sequence by searching for (i) the signature of the PF02556 domain using RPS-BLAST with as threshold an *e*-value of 10^-5^, a score of 35 and a query coverage of 40%, or (ii) TAC (or AC) chaperones potentially not identified by the first request, using an approach described previously (Sala et al., 2013b). This approach allowed the identification of 688 SecB sequences. For each taxonomic group containing SecB sequences, the total number of genomes is given in bold characters and represented by a black frame. The number of genomes containing one or more SecB sequences is given in blue and represented by blue rectangles, and the number of genomes containing TAC or AC systems is depicted in green. **(B)** MCL (Markov Clustering) analysis of the 1981 bacterial sequences contained in the PF02256 conserved domain plus 33 sequences identified previously (Sala et al., 2013b) as TAC chaperones that are absent from the PF02556. A graph was built in which nodes (Circles) correspond to protein sequences and weighted edges represent the BLASTP log(*e*-value) obtained between a pair of proteins. In this case, the homology relationship was inferred when an *e*-value less than or equal to 10^-5^ was observed between two sequences. Partitioning of this graph into communities of highly connected nodes was performed using the MCL program, with an inflat parameter of 1.5. This resulted in 18 groups of SecB sequences, represented by the different colors assigned to the circles in the graph. The highly connected core containing almost all solitary SecB sequences discussed in the text is circled in gray. The three circles with a thick gray line indicate the solitary SecB sequences found outside of the SecB conservation core. MTBC stands for *M. tuberculosis* complex species.

It has been proposed that SecB appeared in the last common ancestor of α-, β-, and γ-proteobacteria and that its conservation is linked to the presence of an outer membrane, and thus an increased need in protein export (van der Sluis and Driessen, 2006). Nevertheless, analysis of the taxonomic repartition of PF02556, the Pfam domain characterizing SecB sequences (http://pfam.sanger.ac.uk/) in a set of 1631 complete and cured bacterial genomes revealed the presence of this domain in seven groups outside proteobacteria. Noticeably, these groups are mainly composed of diderm bacteria, except from the Firmicutes phylum (**Figure 3A**) and in most cases, SecB sequences occur at low frequency when compared to the total number of genomes. This is in sharp contrast with α-, β-, and γ-proteobacteria, where most of the genomes contain at least one SecB sequence (Sala et al., 2013b; **Figure 3A**).

A subset of SecB genes, representing approximately 7.5% of the total number of SecB sequences (52/688), are associated with genes encoding TA systems (or in some cases antitoxin genes alone), as observed for the TAC system of *M. tuberculosis*. This suggests that these putative SecB chaperones might function in the specific control of their cognate TA systems in a manner comparable to that of Rv1957 (Bordes et al., 2011; Sala et al., 2013b). When SecB sequences are present in groups outside of α-, β-, and γ-proteobacteria, they seem to preferentially associate with a TA system (63%, 44/70). In this case, the vast majority of the genomes (>90%) do not possess an additional copy of solitary SecB. Interestingly, TA systems associated with SecB sequences often belong to different families of toxins and/or antitoxins, strongly suggesting that the event of association of a SecB encoding gene with a TA module is a widespread mechanism that occurred several times during evolution (Sala et al., 2013b). The possible involvement of these SecB chaperones in Sec-dependent protein export remains to be determined.

Further analysis on all the bacterial sequences available on the Pfam server for the PF02556 conserved domain (i.e., from both complete and in progress genomes) was performed to study the homology links between SecB sequences using a graph partitioning approach. This revealed that solitary SecB sequences are grouped together in a highly connected core, reflecting a high level of conservation (**Figure 3B**). In this core, several SecB communities (corresponding to the different colors) are well-defined and generally correspond to the taxonomy: the red family contains mainly α-proteobacterial sequences, the dark orange mainly γ-proteobacterial sequences and the light orange mainly β-proteobacterial sequences. Another clearly defined group that emerges from this core, in yellow, contains mainly sequences from *Streptococcus pneumoniae* strains (158/196). The other communities within the core are poorly defined and mainly correspond to other Firmicutes sequences. Most of the TAC (or AC) chaperones are grouped in eight different communities, which seem to have diverged from the solitary SecB core from distinct origins (**Figure 3B**). The light orange group within the core also contains four TA-associated SecB sequences from δ-proteobacteria, thus strongly suggesting a common evolutionary history between TAC chaperones and canonical solitary SecB (Sala et al., 2013b). Yet, in sharp contrast with solitary SecB, the groups of TAC chaperones do not follow the taxonomy and most of them are comprised in regions containing horizontal gene transfer signatures, as it is the case for classical TA systems (Makarova et al., 2009; Sala et al., 2013b).

Paralogs of SecA, SecE, SecY, and SecG are found in Actinobacteria and Firmicutes, either forming a parallel pathway with a dedicated translocon or exploiting the generic Sec translocon to export a specific set of substrates, as it is the case for SecA2 in mycobacteria (Rigel et al., 2009; Sullivan et al., 2012). Interestingly, among the 1631 complete bacterial genomes analyzed 26 of them are predicted to have more than one solitary SecB sequence (up to three in *Acetobacter pasteurianus* IFO 3283-01-42C), eight genomes contain both a solitary and a TA associated SecB, one genome contains two solitary and one TA associated SecB, and two genomes contain two TAC or AC. These additional SecB sequences could function as specialized chaperones for the control of TA systems or for the export of specific substrates, or as generic chaperone induced in response to certain stress conditions. Interestingly, single deletion of *secB1* or *secB2* from *Francisella tularensis subsp. novicida* exhibits a reduced biofilm formation, suggesting that both chaperone paralogs participate in the secretion of specific factors important for the attachment to abiotic surfaces (Margolis et al., 2010). Remarkably, the double *secB* mutant was not viable, suggesting that in the case of *F. tularensis*, SecB chaperones have overlapping functions essential for bacterial survival (Margolis et al., 2010).

## CONCLUDING REMARKS

Extensive genetic and biochemical analyses of SecB chaperone tasking have undoubtedly revealed its key cellular roles as part of the network of generic chaperones that orchestrate proteostasis in *E. coli*. The fact that SecB binds its substrates in a non-native state and prevents their unproductive folding is in agreement with its major role in delivering translocation competent proteins to the inner membrane, as observed for a large number of Sec-dependent presecretory proteins, for the ABC transporter substrate HasA, and perhaps for other proteins whose secretion relies on specific secretion systems that lack dedicated chaperones.

In contrast with the specific and well-described cooperative cascade between SecB and SecA during post-translational targeting of Sec-dependent precursors, the interplay between SecB, TF and DnaK remains poorly understood, and there is a clear lack of knowledge about the substrates that are shared between the three chaperones *in vivo*. In addition, it is not known whether these chaperones actively cooperate to facilitate export of certain proteins, both under normal and stress conditions, and to what extent such cooperation influences early partitioning of newly synthesized proteins. Similarly, it remains to be determined whether some cytosolic proteins or protein complexes do require SecB for their folding and/or assembly, as it is the case for the SecB-like protein Rv1957 and its TA system in *M. tuberculosis*. These are truly open questions that need to be addressed.

The relatively frequent association of SecB proteins with different TA families is intriguing and may reveal interesting new SecB functions, perhaps reflecting a link between toxin activation and membrane jamming. In this respect, an important mechanistic issue will be to determine how TA systems have acquired such a unique addiction for SecB chaperones. Finally, the sporadic presence of solitary SecB-like proteins in monoderm bacteria also suggests novel SecB functions to be discovered.

## Conflict of Interest Statement

The authors declare that the research was conducted in the absence of any commercial or financial relationships that could be construed as a potential conflict of interest.
